# Combined application of arsenic trioxide and lithium chloride augments viability reduction and apoptosis induction in human rhabdomyosarcoma cell lines

**DOI:** 10.1371/journal.pone.0178857

**Published:** 2017-06-02

**Authors:** Sabine B. Schleicher, Julian J. Zaborski, Rosa Riester, Natascha Zenkner, Rupert Handgretinger, Torsten Kluba, Frank Traub, Karen A. Boehme

**Affiliations:** 1 Eberhard Karls University Tuebingen, Children’s Hospital, Department of Hematology and Oncology, Tuebingen, Germany; 2 Eberhard Karls University Tuebingen, Department of Orthopedic Surgery, Laboratory of Cell Biology, Tuebingen, Germany; 3 Eberhard Karls University Tuebingen, Department of Orthopedic Surgery, Tuebingen, Germany; Universite Paris-Sud, FRANCE

## Abstract

Rhabdomyosarcomas (RMS) are the most prevalent soft tissue sarcomas affecting children and adolescents. Despite intensive treatment consisting of multimodal chemotherapy and surgery RMS patients diagnosed with metastatic disease expect long term survival rates of only 20%. Often multidrug resistance arises upon initial response emphasizing the need for new therapeutic drugs to improve treatment efficiency. Previously, we demonstrated the efficacy of the FDA approved drug arsenic trioxide (ATO) specifically inhibiting viability and clonal growth as well as inducing cell death in human RMS cell lines of different subtypes. In this study, we combined low dose ATO with lithium chloride (LiCl), which is approved as mood stabilizer for the treatment of bipolar disorder, but also inhibits growth and survival of different cancer cell types in pre-clinical research. Indeed, we could show additive effects of LiCl and ATO on viability reduction, decrease of colony formation as well as cell death induction. In the course of this, LiCl induced inhibitory glycogen synthase kinase-3β (GSK-3β) serine 9 phosphorylation, whereas glioma associated oncogene family 1 (GLI1) protein expression was particularly reduced by combined ATO and LiCl treatment in RD and RH-30 cell lines, showing high rates of apoptotic cell death. These results imply that combination of ATO with LiCl or another drug targeting GSK-3 is a promising strategy to enforce the treatment efficiency in resistant and recurrent RMS.

## Introduction

Rhabdomyosarcomas (RMS) are the most prevalent soft tissue tumors in children and adolescents, accounting for about 5% of all pediatric tumors [1, 2]. Estimated 350 new cases of RMS are diagnosed each year in patients under 20 years of age in the United States [1].

Today, chemotherapeutic treatment of RMS includes vincristine, actinomycin D and cyclophosphamide (VAC). Besides, in some protocols doxorubicin is administered. For treatment of patients with metastatic RMS, two additional drugs, etoposide and ifosfamide (IE) are added [3–5]. However, multidrug resistance often arises upon initial response [6, 7]. Therefore, new targeted therapies are urgently needed to improve treatment efficiency in RMS [8].

Recently, we showed that the FDA approved drug arsenic trioxide (ATO, As_2_O_3_) effectively reduced viability and induced cell death in RMS cell lines of embryonal (ERMS), alveolar (ARMS) and sclerosing, spindle cell subtype. Moreover, combination of the glioma-associated oncogene family (GLI) inhibitior ATO with itraconazole, which targets smoothened (SMO), another component of the hedgehog (Hh) pathway, potentiated the reduction of colony formation [9].

Other pathways implicated in RMS biology are the phosphatidylinositol 3-kinase (PI3K)-protein kinase B (PKB) pathway [10, 11] and the Wnt-β-catenin pathway [12]. Both pathways converge on the highly conserved serine/threonine kinase glycogen synthase kinase-3 (GSK-3) exhibiting constitutive activity [13]. The two isoforms of GSK-3, GSK-3α and GSK-3β have redundant but also distinct functions in cellular metabolism, proliferation and differentiation. Phosphorylation at serine 9 (GSK-3β) or serine 21 (GSK-3α) inhibits the kinase activity by induction of a conformational change, which can nevertheless be overcome by high substrate concentrations [13].

Zeng et al. demonstrated that GSK-3 inhibition using different compounds inhibited proliferation and induced apoptosis in the ARMS cell line RH-30 more efficiently compared to the ERMS cell line RD, which was associated with reduced transcriptional activity of the paired box 3/ forkhead transcription factor (PAX3-FKHR) in RH-30 cells [14]. Lithium chloride (LiCl) is used as a mood stabilizer for treatment of bipolar disorder for over 60 years [15]. Inhibition of GSK-3 kinase activity is mediated by competition of lithium ions with magnesium [16]. Moreover, inhibition of GSK-3 by LiCl can be evidenced by an increase of phosphorylation [17], which is due to PKB activation [18]. LiCl inhibits proliferation and induces apoptosis in several cancer types including glioma, colon adenocarcinoma, medulloblastoma, hepatocellular carcinoma, pancreatic adenocarcinoma and sarcoma *in vitro* and *in vivo* [19–24].

In addition to GSK-3, LiCl also inhibits Hh signaling at the level of GLI. In pancreatic cancer cells it has been shown that lithium treatment reduced the GLI1 mRNA and protein expression [23]. This effect was reproduced in primary murine medulloblastoma spheres, where LiCl reduced GLI1 abundance whereas the repressor form of GLI3 was upregulated, which was accompanied by a G2/M cell cycle arrest and induction of a senescent-like state [17]. Moreover, a direct interaction of stabilized β-catenin with GLI1 has been shown, which was increased under lithium treatment mediating GLI1 degradation [17].

Huang et al. revealed redundant induction of GSK-3β serine 9 phosphorylation in A431 epidermoid carcinoma cells and HaCaT immortalized keratinozytes by both ATO and LiCl leading to GSK-3β inactivation [25].

In this study, we show additive effects of LiCl and ATO on viability reduction, decrease of colony formation, as well as cell death induction. The viability reduction observed in monolayer cultures could be confirmed in 3D cultures and caspase activation indicated apoptosis initiation. In the course of this, LiCl induced inhibitory GSK-3β serine 9 phosphorylation, whereas GLI1 protein expression was reduced by combined ATO and LiCl treatment in RD and RH-30 cell lines, showing high rates of apoptotic cell death.

## Materials and methods

### Reagents

ATO (Trisenox, Pharmacy of University Hospital Tuebingen) and LiCl (Sigma-Aldrich, Taufkirchen, Germany) were dissolved in deionised water. For cell culture treatment stock solutions were further diluted in culture medium.

### Cell lines and culture

RD cells were purchased from CLS Cell Lines Service GmbH (Eppelheim, Germany). RH-30 cells were obtained from ATCC (Manassas, VA, USA). SRH (sclerosing spindle cell RMS) were established and characterized at the University Children’s Hospital Tuebingen. The patient has given her written informed consent to the scientific analysis and cell line establishment from tissue samples, the study of which was approved by the ethics committee of the University of Tuebingen (008/2014BO2). Normal skeletal muscle cells from adult donors (SKMC) were purchased from PromoCell (Heidelberg, Germany). All cell lines were maintained in Dulbecco’s modified Eagle’s medium (DMEM) with GlutaMAX, 4.5 g/l D-glucose (Gibco, Life Technologies, Darmstadt, Germany) supplemented with 10% FCS (Biochrom, Berlin, Germany) at 37°C in humidified atmosphere containing 5% CO_2_.

### Cytotoxicity assay

CellTiter 96^®^ AQueous One Solution Cell Proliferation (MTS) Assay (Promega, Mannheim, Germany) was used to measure cell viability via redox enzyme activity, according to the protocol provided by the manufacturer. Each 0.5 × 10^4^ cells/well RD, RH-30 and SKMC or 1.5 × 10^4^ cells/well SRH were grown in 96-well plates. 24 h after seeding, the cells were incubated in the presence of ATO, LiCl or inhibitor combinations for another 96 h at 37°C in a humidified atmosphere of 5% CO_2_ in air. At the end of the incubation period, MTS reagent was added to the wells, and the plate was incubated for 1.5 h protected from light. Absorbance was recorded at 490nm with a reference wavelength of 630 nm using an EL 800 reader (BioTek, Winooski, VT, USA).

### IC_50_ determination

IC_50_ values of LiCl and ATO were determined for the different cell lines by nonlinear regression using GraphPad Prism V6.0 software (La Jolla, CA, USA).

### Colony formation assay

The colony formation assay was performed according to Franken et al. [26]. In detail, RD and RH-30 cells were plated at a density of 0.5 × 10^3^ cells/well in a 6-well plate and incubated with increasing concentrations of ATO, LiCl or inhibitor combinations for 72 h. After 10 days subsequent growth in standard growth medium, cells were fixed using ice cold methanol for 15 min, washed and stored in PBS. Visualisation of fixed cell colonies was achieved by incubating the cells with 0.5% (w/v) crystal violet for 30 min. Excess crystal violet was removed by washing with PBS. Visible colonies, consisting of ≥ 50 cells, were counted. The colony formation rate was determined: (number of colonies/number of plated cells) x 100.

### Spheroid assay

For generation of 3D spheroids [27], 0.5 x 10^4^ cells of the RMS cell lines RD and RH-30 were seeded in ultra-low-attachment, U-bottom 96 well plates (Thermo Scientific, Rochester, NY, USA). After 96 h spheroid formation was documented by micrographs and ATO and LiCl were added to the culture medium as indicated. Six days later a second documentation by micrographs was performed. CellTiter-Blue^®^ viability assay (Promega, Mannheim, Germany) was used to determine spheroid viability at day 6 via redox enzyme activity, according to the protocol provided by the manufacturer. Fluorescence intensity was measured at a 530 nm excitation wavelength and a 590 nm emission wavelength using a Wallac 1420 Victor2 multilabel counter (Perkin Elmer, Waltham, MA, USA).

### PathScan^®^ sandwich ELISA assay

0.5 x 10^6^ RD, SRH, RH-30 and SKMC cells were incubated with inhibitor concentrations indicated for 36 h in 6-well-plates and the PathScan^®^ ELISA (Cell Signaling Technology, Leiden, Netherlands) specific for phospho-GSK-3β (serine 9) ♯7311C and total GSK-3β ♯7265C was carried out according the manufacturer’s instructions. Absorbance was recorded at 450 nm using an EL 800 reader.

### Western blot analysis

Each 2.5 x 10^5^ SRH, RD and RH-30 cells or SKMC were incubated in 6-well plates with inhibitor concentrations indicated for 36 h. For analysis, cells were washed with PBS and lysed in protein lysis buffer (40 mM Tris/HCl pH7.4, 300 mM NaCl, 2 mM EDTA, 20% glycerol, 2% Triton X-100) supplemented with proteinase inhibitor at 4°C. Insoluble material was removed by centrifugation. The protein concentration in the supernatant was determined by Bradford protein assay. 40 μg of the protein samples were separated by 10% SDS-PAGE and transferred to a hydrophobic polyvinylidene difluoride (PVDF) membrane (Immobilon-P, Merck KGaA, Darmstadt, Germany). After blocking with 5% powdered milk (Carl Roth, Karlsruhe, Germany) in TBS-T, membranes were incubated with primary antibodies [GLI1 rabbit mAb ♯2553 1:1000, PCNA (PC10) mouse mAb ♯2586 1:2000, both Cell Signaling Technology, Leiden, Netherlands] with gentle shaking overnight at 4°C according the manufacturers protocols. Membranes were washed three times with TBS-T. Secondary antibody (horseradish peroxidase-conjugated anti-rabbit pAb or horseradish peroxidase-conjugated anti-mouse pAb, both 1:10000, Jackson Immuno Research, West Grove, PA, USA) were added for 2 h, and the membranes were washed another three times with TBS-T. Proteins were detected using ECL Western Blotting Substrate (Thermo Scientific, Waltham, MA, USA) with membranes exposed to Amersham Hyperfilm ECL (GE Healthcare, Pittsburgh, PA, USA). A pre-stained protein ladder (PageRuler Plus; Thermo-Scientific, Waltham, MA, USA) was used for determination of molecular weights. Image J (NIH) was utilized for western blot quantification.

### Flow cytometry

Membrane integrity as indicator for cell death was determined using the fixable viability dye eFluor^®^ 450 (eBioscience, San Diego, CA, USA). 2,5 x 10^5^ SRH, RD, RH-30 and SKMC cells were incubated with inhibitor concentrations indicated for 72 h, washed with PBS, detached with trypsin, and stained for 30 min at 4°C in the dark. Cells were washed with PBS and fixed with 0.5% formaldehyde diluted in PBS before being resuspended in FACS buffer (PBS containing 2% FCS, 2 mM EDTA). Flow-cytometric analysis was performed on a LSRII flow cytometer (Becton Dickinson, Franklin Lakes, NJ, USA) using the violet laser (405 nm) for excitation and a 450/50 band pass filter for detection. FlowJo Software (Tree Star Inc., Ashland, OR, USA) was utilized for data evaluation.

### Caspase assay

Apoptosis induction was measured in SRH, RD, RH-30 and SKMC using the Apo-ONE^®^ homogenous caspase 3/7 assay (Promega, Mannheim, Germany) according to the protocol provided by the manufacturer. Each 1 × 10^4^ cells/well RD and RH-30 cells, 0.5 × 10^4^ cells/well SKMC cells or 3 × 10^4^ cells/well SRH cells were grown in black 96-well plates with opaque bottom. 24 h after seeding, the cells were incubated in the presence of ATO, LiCl or inhibitor combinations for another 36 h at 37°C in a humidified atmosphere of 5% CO_2_ in air. At the end of the incubation period, the Z-DEVD-R110 substrate was added to the wells, and the plate was incubated for 1 h protected from light. Fluorescence was recorded at excitation wave length 485 nm, emission wave length 528 nm with a Wallac 1420 Victor2 multilabel counter.

### Statistical analysis

All statistical tests were performed using GraphPad Prism V6.0 software and statistical differences were analysed by two-way ANOVA. Multiple comparisons between groups were performed using Tukey's test. Significant differences between groups (p ≤ 0.05, p ≤ 0.01, p ≤ 0.001 or p ≤ 0.0001) are indicated by small letters. Multiplicity adjusted p values for each treatment against mock treated control and p values for combined treatment relative to single treatment were determined.

Drug interactions were analyzed by the combination index (CI) method based on that described by Chou [28] using CalcuSyn software (Biosoft, Cambridge, UK). CI < 0.9 indicates synergism, 0.9–1.1 additivity and >1.1 antagonism.

## Results

### Arsenic trioxide in combination with lithium chloride reduces viability of human RMS cell lines

MTS viability assays were performed to determine IC_50_ values for LiCl in the RMS cell lines SRH, RD and RH-30 as well as SKMC (Table 1, S1 Fig). The IC_50_ values obtained for the RMS cell lines ranged from 20.26 mM in RH-30 to 32.14 mM in SRH, whereas SKMC were less sensitive (50.12 mM).

**Table 1:** LiCl reduces viability in human RMS cell lines. MTS assays were performed four days after treatment with LiCl in three RMS cell lines and SKMC in quadruplicate. Mock treated control was set to 100% viability. IC_50_ values were determined by nonlinear regression of MTS results using GraphPad Prism 6.

Inhibitor	Cell line	IC_50_
LiCl	SKMC	50.12 mM
	SRH	32.14 mM
	RD	25.97 mM
	RH-30	20.26 mM

**Fig 1:**
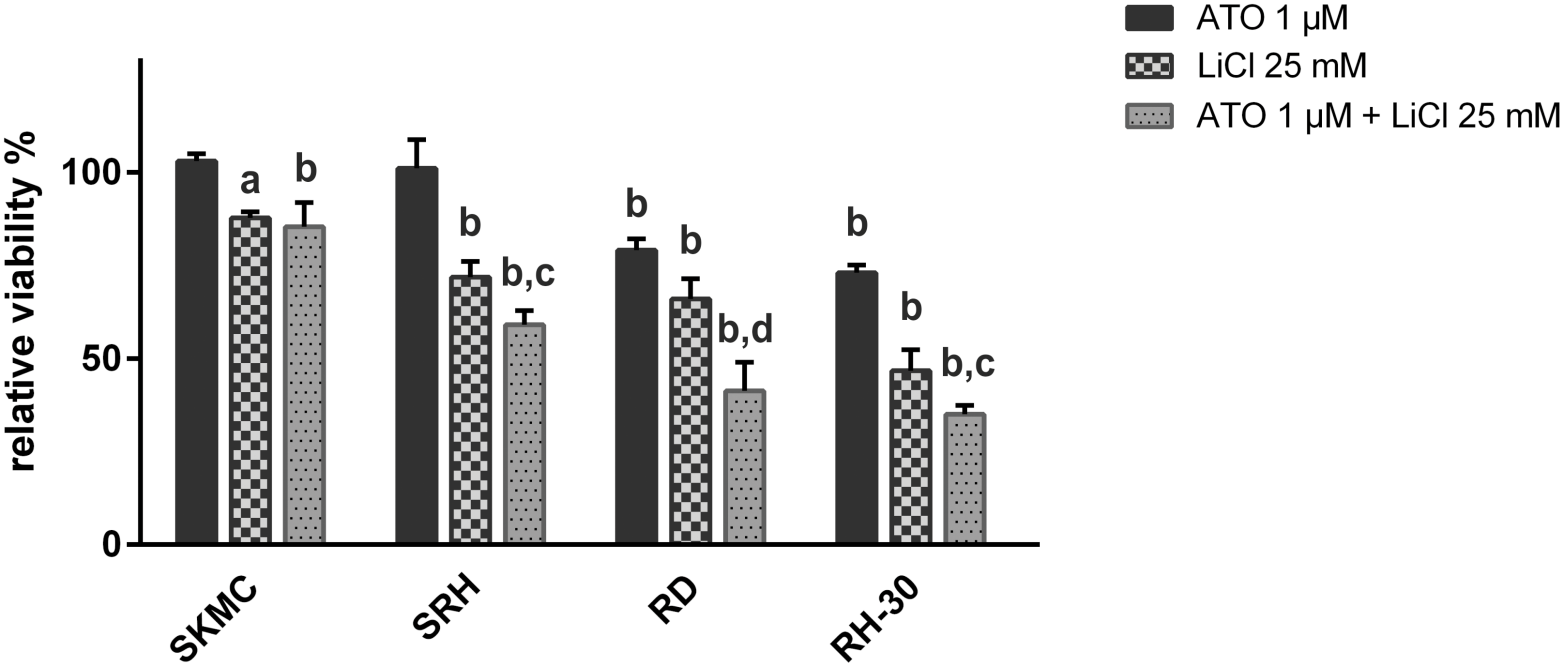
ATO in combination with LiCl enhances viability reduction in human RMS cell lines. MTS assays were performed four days after single or combined treatment with 1 μM ATO and 25 mM LiCl in three RMS cell lines and SKMC in quadruplicate. Mock treated control was set to 100% viability. Error bars indicate the standard deviation. Significant differences between groups are indicated by small letters. Multiplicity adjusted p values for each treatment against mock treated control were determined: “a” indicates p ≤ 0.001, “b” indicates p ≤ 0.0001. In addition, p values for combined treatment relative to single treatment were determined: “c” indicates p ≤ 0.01, “d” indicates p ≤ 0.0001.

IC_50_ values for ATO have been obtained earlier [9] (S2 Fig). To analyse potential additive effects of ATO in combination with LiCl, drug concentrations in the range of the IC_50_ values were applied in MTS viability assays. Combination of 1 μM ATO and 25 mM LiCl significantly enhanced viability reduction compared to single treatment in SRH, RD and RH-30 cells (Fig 1). Additive effects could be confirmed by calculation of the combination index (CI) as described in the materials and methods section in SRH (CI 1,08), RD (CI 1.07) and RH-30 cells (CI 0.92). Viabilty of SKMC came not below 85 ± 6.5% obtained with the combination of 1 μM ATO and 25 mM LiCl.

### Arsenic trioxide, lithium chloride, and combinations thereof reduce colony formation of human RMS cell lines

**Fig 2:**
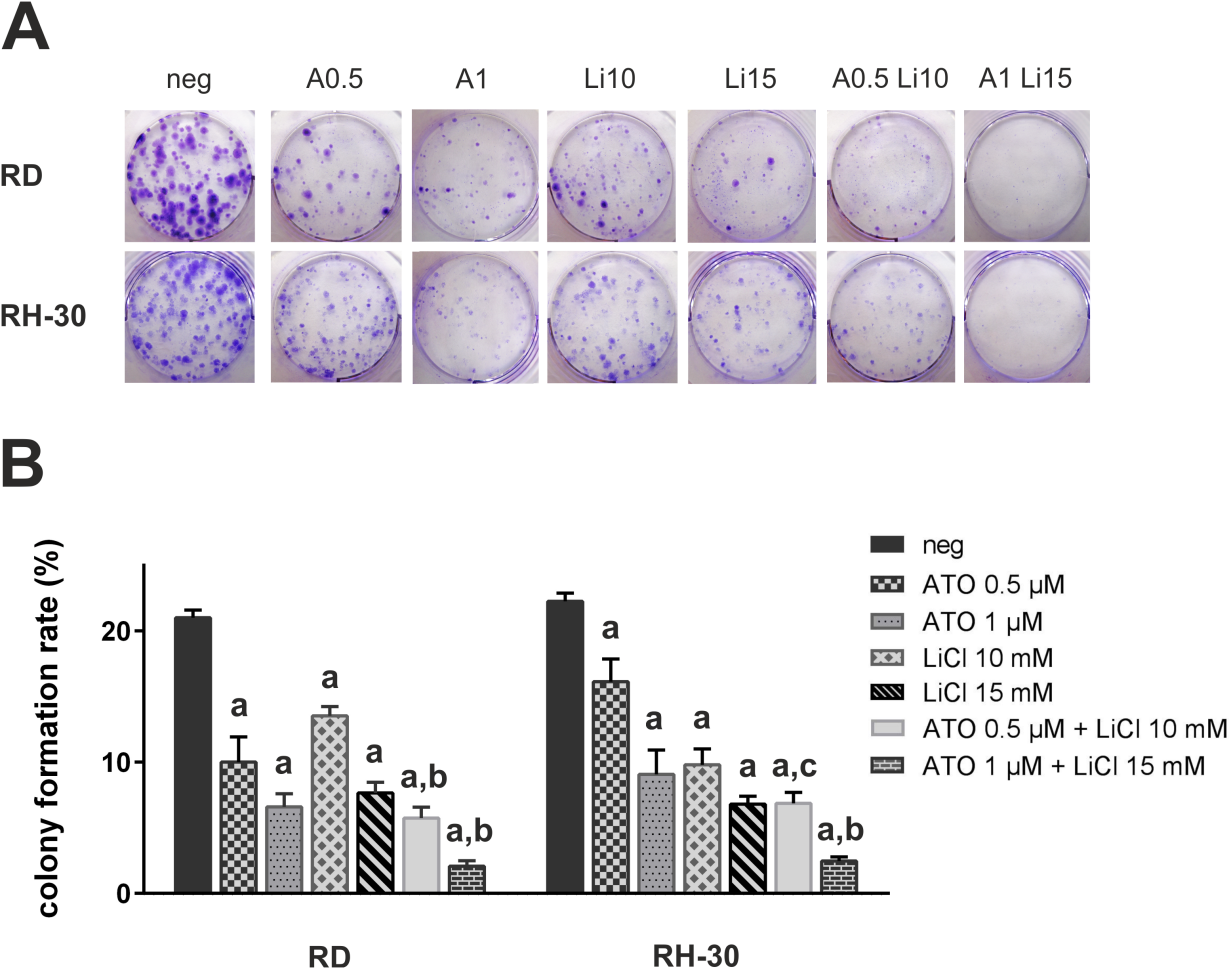
ATO in combination with LiCl impairs colony formation of human RMS cell lines. 0.5 × 10^3^ RD or RH-30 cells were seeded in 6-well plates and incubated with 0.5 μM (A0.5) or 1 μM (A1) ATO, respectively 10 mM (Li10) or 15 mM (Li15) LiCl for 72 h. Subsequently, cells were incubated in normal culture medium for another ten days. (A) Visualisation of fixed cell colonies was achieved by incubating the cells with 0.5% (w/v) crystal violet. Representative images for each cell line are shown. (B) Colonies of three independent experiments were counted. The graph indicates the mean colony formation rates with standard deviations. Significant differences between groups are indicated by small letters. Multiplicity adjusted p values for each treatment against mock treated control were determined: “a” indicates p ≤ 0.0001. In addition, p values for combined treatment relative to single treatment were determined: “b” indicates p ≤ 0.001, “c” indicates p ≤ 0.01.

Additive reduction of colony formation after combined treatment with ATO and itraconazole has been shown before for the RMS cell lines RD, RUCH3, RH-30 and ZF [9]. Fig 2A and 2B demonstrate that already 0.5 μM ATO significantly reduced colony formation in RD and RH-30 cells. Moreover single doses of 10 mM LiCl substantially decreased colony formation rates in both cell lines. Escalation of the LiCl and ATO doses to 15 mM, respectively 1 μM led to a decline of the colony formation rate below 10%. Drug combinations of 0.5 μM ATO with 10 mM LiCl and 1 μM ATO with 15 mM LiCl were both significantly more efficient compared to single treatment. In addition to a massive reduction of colony numbers, also the size of remaining colonies decreased. The CI value for the combination of 0.5 μM ATO with 10 mM LiCl in RD cells was 0.98, indicating additivity. Combination of 1 μM ATO with 15 mM LiCl resulted in a synergistic CI of 0.80. In RH-30 cells, the lower dose combination generated a CI of 1.17, indicating slight antagonism, whereas the higher drug doses led to an additive reduction of colony formation (CI 1.02).

### Arsenic trioxide, lithium chloride and their combination reduce viability and integrity of human RMS 3D spheroid cultures

**Fig 3:**
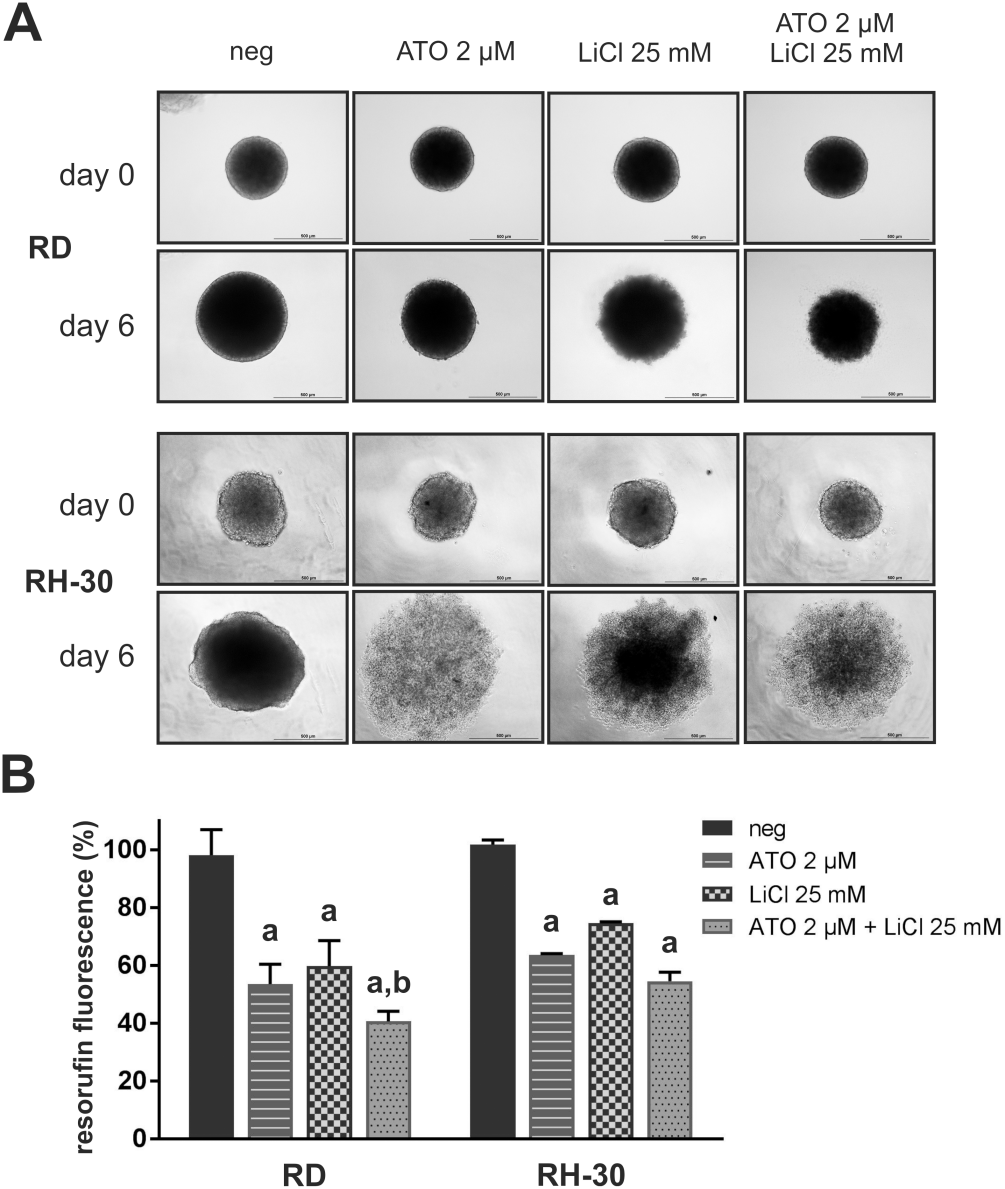
ATO, LiCl and drug combinations reduce RMS cell viability and spheroid integrity in 3D cultures. Each 0.5 × 10^4^ RD or RH-30 cells were plated in non-adherent, U-bottom 96 well plates. (A) After four days spheroid formation was documented at the microscope (day 0). Spheroids were incubated with 2 μM ATO and 25 mM LiCl as indicated for another six days whereupon a second micrograph was taken (day 6). Representative micrographs of three independent experiments are shown. (B) Resazurin viability assays were performed at day 6 and resorufin fluorescence of mock treated control was set to 100% viability. Error bars indicate the standard deviation. Significant differences between groups are indicated by small letters. Multiplicity adjusted p values for each treatment against mock treated control were determined: “a” indicates p ≤ 0.0001. In addition, p values for combined treatment relative to single treatment were determined: “b” indicates p ≤ 0.05.

To simulate gradients in nutrient and oxygen availability as well as drug penetration *in vivo* we used 3D spheroid cultures of RD and RH-30 cells (Fig 3A and 3B). 2 μM ATO, 25 mM LiCl as well as the combination reduced growth of RD spheroids. Moreover, LiCl and the combination of LiCl and ATO compromised RD spheroid surface integrity. In contrast, RH-30 spheroid cultures appeared bloated and completely lost integrity upon all drug treatments. To quantify the effect of ATO and LiCl in 3D cultures, resazurin assays were performed after taking pictures at day 6 (Fig 3B). Indeed, both single treatments and the combination of ATO and LiCl significantly impaired cell viability in spheroids of both cell lines. However, only in RD spheroids the impact of the combination was significantly higher compared to both single treatments, whereas in RH-30 spheroids the combination clearly outranged LiCl, but not ATO.

### Arsenic trioxide in combination with lithium chloride intensifies cell death induction in human RMS cell lines

**Fig 4:**
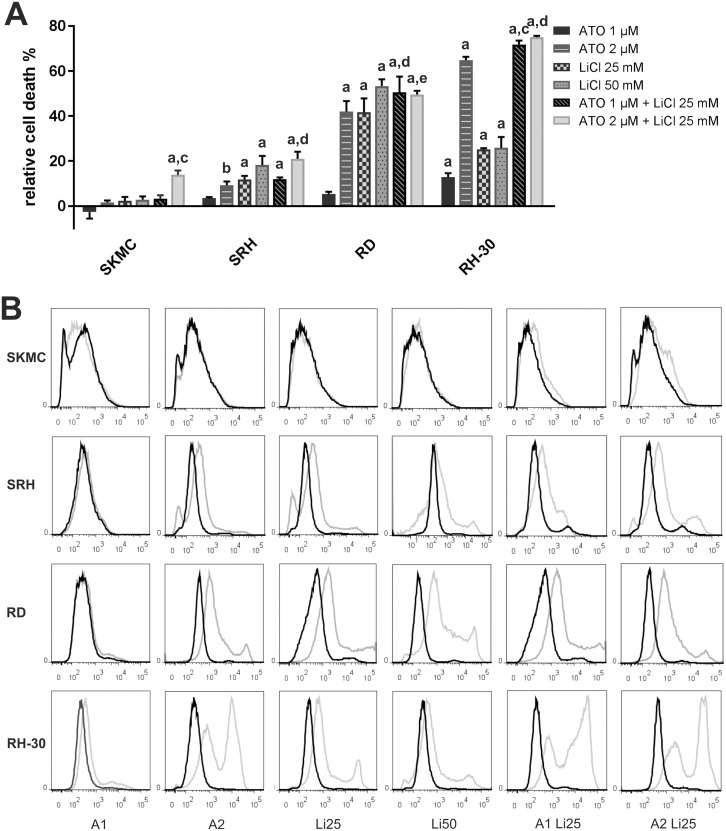
Combination of ATO and LiCl enhances cell death in human RMS cell lines. Incorporation of the fixable viability dye eFluor^®^ 450 was analysed by flow cytometry three days after single and combined treatment with 1 μM (A1) or 2 μM (A2) ATO and 25 mM (Li25) or 50 mM (Li50) LiCl in three RMS cell lines and SKMC in triplicate. (A) Mock treated control was set to zero % cell death. Error bars indicate the standard deviations. Significant differences between groups are indicated by small letters. Multiplicity adjusted p values for each treatment against mock treated control were determined: “a” indicates p ≤ 0.0001, “b” indicates p ≤ 0.01. In addition, p values for combined treatment relative to single treatment were determined: “c” indicates p ≤ 0.0001, “d” indicates p ≤ 0.001, “e” indicates p ≤ 0.01. (B) For all treatments (grey curves) and corresponding mock treated controls (black curves) representative FACS histogram plots are shown.

As significant impairment of viability was already achieved with doses of 1 μM ATO and 25 mM LiCl, whereas prevention of 3D spheroid culture growth required 2 μM ATO in combination with 25 mM LiCl, both combinations were tested for their impact on cell death induction, examined by incorporation of the fixable viability dye eFluor^®^ 450 (Fig 4A and 4B). Indeed, already the combination using 1 μM ATO was sufficient to induce 50.5 ± 7% cell death in RD and 71.8 ± 1.7% cell death in RH-30 cells, which was significantly more efficient compared to single treatment in both cell lines. However, in RD cells also 25 mM LiCl alone was effective (41.7 ± 6.1%), whereas only 25.2 ± 0.6% of the LiCl treated RH-30 were eFluor^®^ 450 positive. In SRH cells the cell death obtained using 25 mM LiCl (11.8 ± 1.8%) was the same as after the combination with 1 μM ATO (11.9 ± 0.8%), whereas in SKMC the maximum incorporation of eFluor^®^ 450 was 3.2 ± 1.6% after treatment with the drug combination. Doubling of the ATO dose to 2 μM strongly enhanced dye incorporation in RD (42 ± 4.7%) and RH-30 cells (64.8 ± 1.5%), whereas cell death after application of the combination stagnated in RD cells at 49.6 ± 1.6% and RH-30 cells at 75 ± 0.6%, which was still significantly higher compared to single application. In the more resistant SRH cells the combination with 2 μM ATO induced 21 ± 3.1% cell death, significantly more compared to both single treatments and nearly twice as much as with the lower dose combination. Indeed, the combination of 2 μM ATO and 25 mM LiCl also perceptibly affected SKMC with 13.9 ± 1.9% eFluor^®^ 450 positive cells. Application of 50 mM LiCl resulted in 18.2 ± 4% eFluor^®^ 450 positive SRH cells, whereas 53.4 ± 4% of the RD cells and 25.9 ± 5% of the RH-30 cells incorporated the dye. The CI was calculated for all combinations showing significant effects. Indeed, in SRH cells, the combination of 2 μM ATO and 25 mM LiCl was slightly antagonistic (CI 1.20), whereas in RD cells combination of 1 μM ATO with 25 mM LiCl was additive (CI 0.98), but increasing the ATO dose to 2 μM was antagonistic (CI 1.42). In RH-30 cells both drug combinations utilizing 1 μM ATO (CI 0.37) or 2 μM ATO (CI 0.56) were clearly synergistic. In addition, the effect of the combination of 2 μM ATO and 25 mM LiCl in SKMC cells was also synergistic (CI 0.20) considering the lack of reaction upon all other treatments.

### Arsenic trioxide in combination with lithium chloride enhances caspase activation in human RMS cell lines

**Fig 5:**
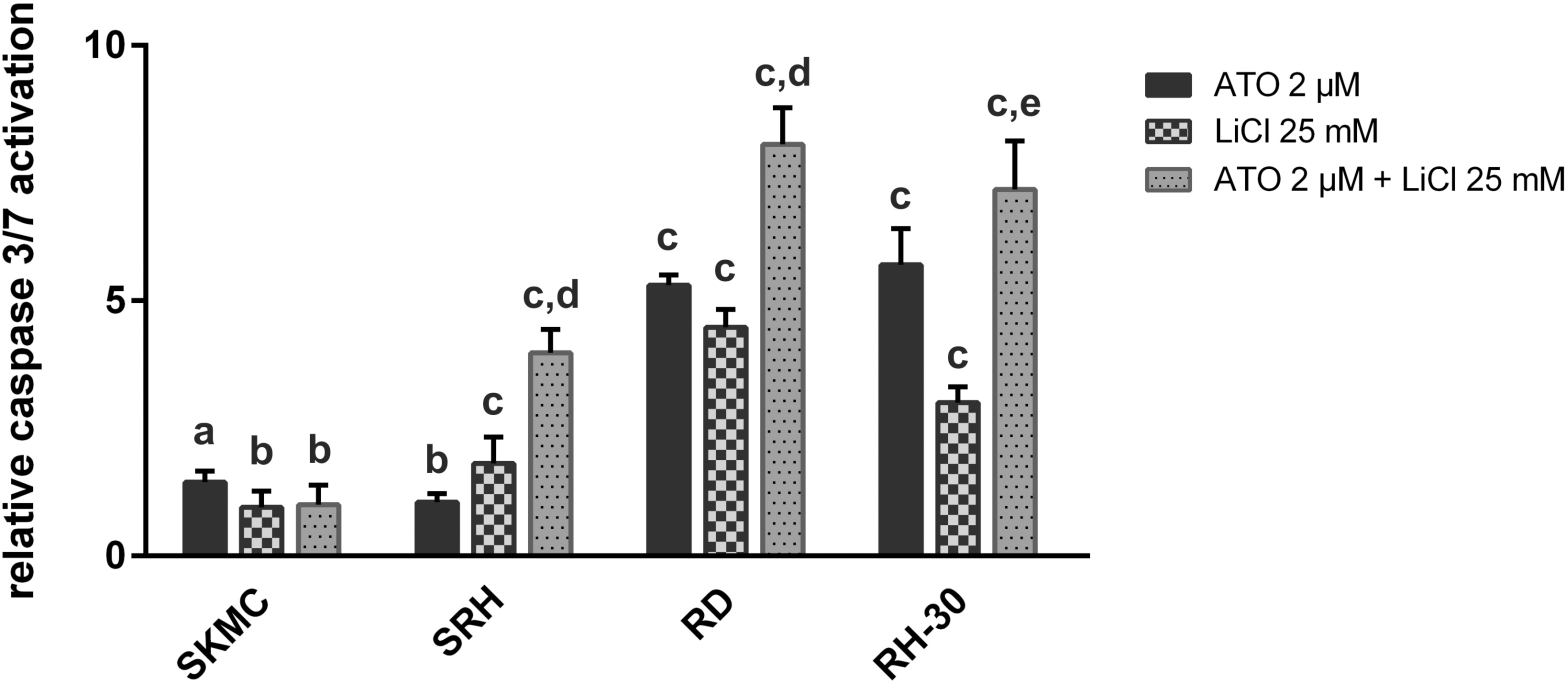
ATO in combination with LiCl enhances caspase activation in human RMS cell lines. Caspase assays were performed 36 h after single or combined treatment with 2 μM ATO and 25 mM LiCl in three RMS cell lines and SKMC in quadruplicate. Caspase activity of mock treated control was set to zero. Error bars indicate the standard deviation. Significant differences between groups are indicated by small letters. Multiplicity adjusted p values for each treatment against mock treated control were determined: “a” indicates p ≤ 0.001, “b” indicates p ≤ 0.05, “c” indicates p ≤ 0.0001. In addition, p values for combined treatment relative to single treatment were determined: “d” indicates p ≤ 0.0001, “e” indicates p ≤ 0.01.

To distinguish apoptotic from necrotic cell death a caspase 3/7 assay was performed, indicating significant enhancement of caspase activation by a combination of 2 μM ATO with 25 mM LiCl in SRH, RD and RH-30 cells (Fig 5). However, maximum caspase activation in SRH cells was only 4 ± 0.5 fold, compared to 8.1 ± 0.7 fold in RD and 7.2 ± 1 fold in RH-30, which was also reflected in a lower cell death rate in SRH compared to RD and RH-30 cells. Maximum caspase activation obtained with 2 μM ATO in SKMC was 1.4 ± 0.2 fold, indicating specific caspase activation in the RMS cell lines.

### Lithium chloride induces inhibitory GSK-3β phosphorylation in human RMS cell lines

**Fig 6:**
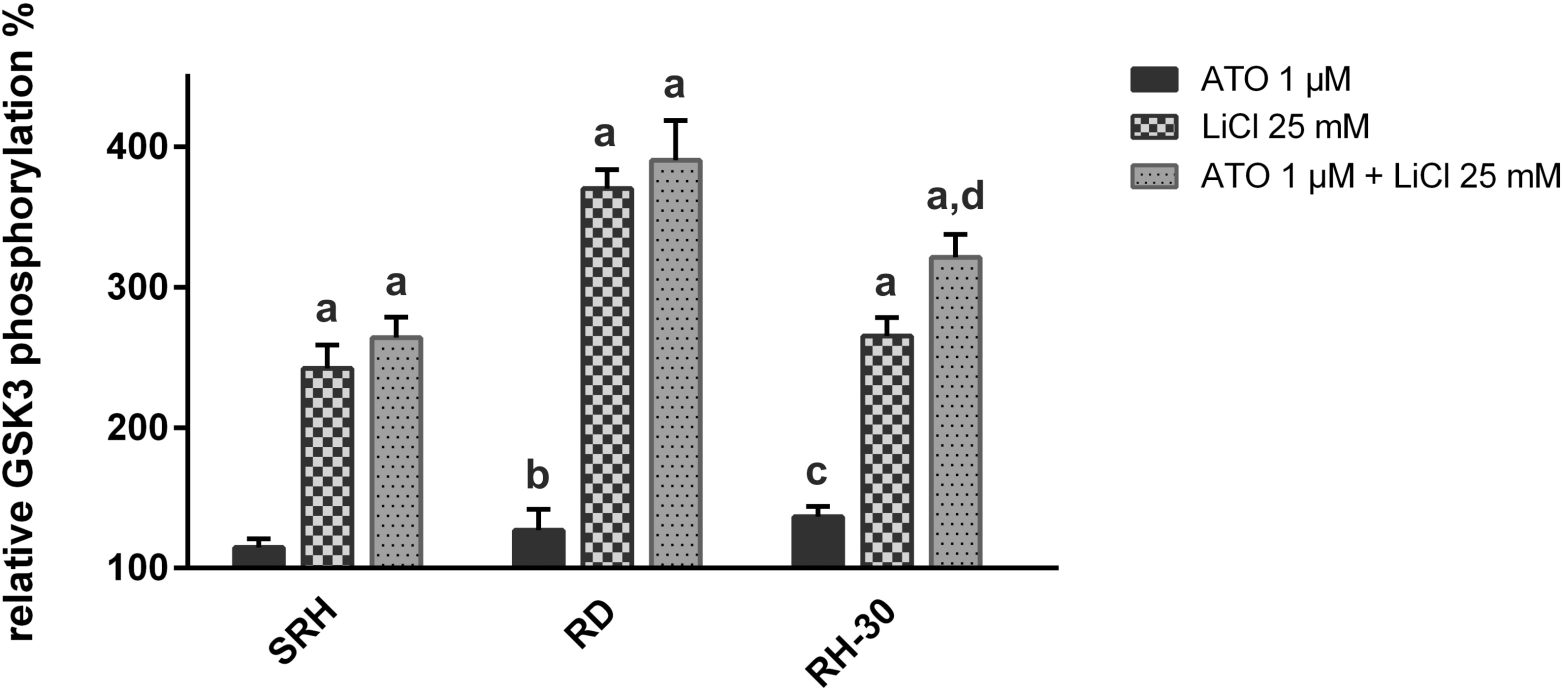
LiCl enhances inhibitory GSK-3β phosphorylation in human RMS cell lines. PathScan^®^ ELISA assays detecting total and phospho-GSK-3β (serine 9) were performed after 36 h of single or combined treatment with 1 μM ATO and 25 mM LiCl in three RMS cell lines in quadruplicate. Mock treated control was set to 100% GSK-3β abundance, respectively phosphorylation. The relative phosphorylation was calculated by normalizing GSK-3β phosphorylation to total GSK-3β. Error bars indicate the standard deviation. Significant differences between groups are indicated by small letters. Multiplicity adjusted p values for each treatment against mock treated control were determined: “a” indicates p ≤ 0.0001, “b” indicates p ≤ 0.05, “c” indicates p ≤ 0.01. In addition, p values for combined treatment relative to single treatment were determined: “d” indicates p ≤ 0.0001.

To elucidate changes in cellular signaling implicated in impaired proliferation and enhanced cell death, GSK-3β abundance and inhibitory serine 9 phosphorylation were investigated using ELISA assays (Fig 6). Whereas GSK-3β expression was steady under single and combined treatment using 1 μM ATO and 25 mM LiCl, LiCl alone and in combination with ATO induced a massive increase of GSK-3β serine 9 phosphorylation in SRH, RD and RH-30 cells. The strongest response was observed in RD cells, followed by RH-30 cells, where a combination with ATO still significantly reinforced serine 9 phosphorylation compared to single treatment.

### Arsenic trioxide and lithium chloride affect GLI1 abundance in human RMS cell lines

**Fig 7:**
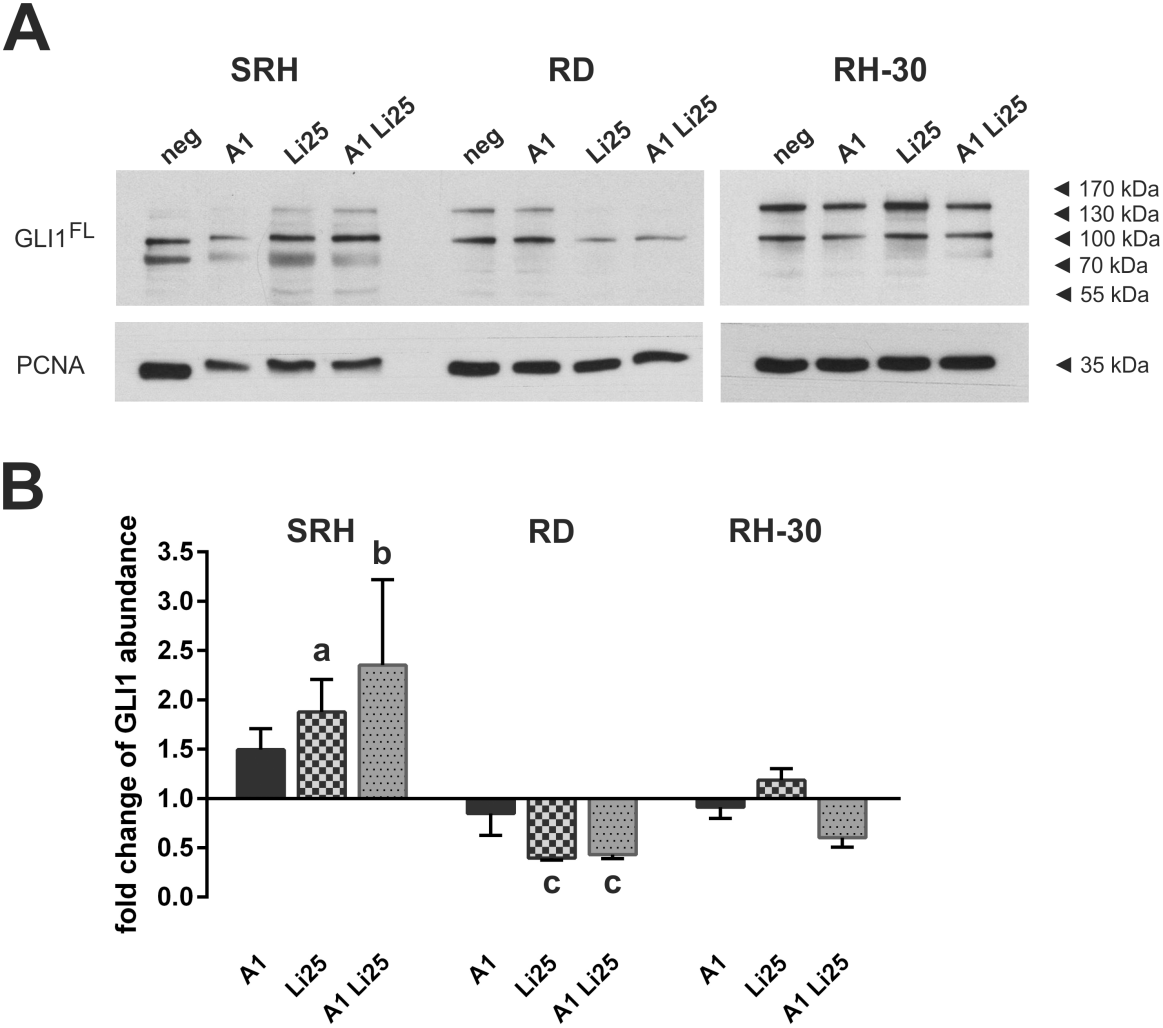
ATO and LiCl affect GLI1 abundance in human RMS cell lines. (A) Western blot analysis with antibodies against GLI1 and PCNA as loading control was performed after 36 h of single or combined treatment with 1 μM ATO and 25 mM LiCl in three RMS cell lines in triplicate. (B) Signals of three independent experiments were quantified. The graph shows the mean values and standard deviations of full length GLI1 abundance after treatment with ATO and LiCl compared to mock treated control. Significant differences between groups are indicated by small letters. Multiplicity adjusted p values for each treatment against mock treated control were determined: “a” indicates p ≤ 0.01, “b” indicates p ≤ 0.0001, “c” indicates p ≤ 0.05.

Reduction of GLI1 protein abundance by treatment with 5 μM ATO has been shown for the RMS cell lines RD and RH-30, whereas SRH cells were the only RMS cell line investigated showing increased GLI1 expression upon ATO administration [9]. Using 1 μM ATO, in SRH cells already an induction of the GLI1 protein could be observed (Fig 7A and 7B, S3 Fig), whereas GLI1 expression was slightly attenuated in RD and RH-30 cells. 25 mM LiCl significantly reduced GLI1 expression in RD cells, while it was slightly enhanced in RH-30 cells. Again, SRH cells showed an obviously increased expression. The combination of 1 μM ATO and 25 mM LiCl reduced the GLI1 protein expression both in RH-30 and RD cells, yet expression was particularly upregulated in SRH cells.

## Discussion

Despite intensive, multimodal chemotherapy long term survival rates of patients with metastatic RMS are only about 20% [5]. Often multidrug resistance arises upon initial response [6, 7], emphasizing the need for new therapeutic drugs to improve treatment efficiency in RMS [8].

Aberrant activation of the Hh pathway, but also of signaling cascades involving GSK-3 have been shown for several cancers including RMS [11–13, 29]. Inhibition of Hh and GSK-3 pathways reduces proliferation and induces apoptotic cell death of tumor cells *in vitro* and *in vivo* [9, 10, 23, 24].

We previously demonstrated that ATO mediated inhibition of viability and colony formation as well as cell death induction in human ERMS and ARMS cell lines is associated with the reduction of GLI1 protein abundance [9]. ATO is approved by the FDA for first line treatment of acute promyelocytic leukemia (APL) [30]. Moreover, clinical trials for the use of ATO in different solid malignancies have been conducted or are ongoing. Although, the application of ATO as single agent was often disappointing, combination with other drugs appears to be promising [31]. In order to administer physiologically easily tolerable doses, in this study we used 0.5 to 2 μM ATO and combined it with 10 to 25 mM LiCl. Admittedly, the therapeutic range of lithium serum concentrations for long term treatment of patients with bipolar disorder is 0.4–1.5 mM [15, 32]. However, in cancer research the short term *in vitro* use of LiCl doses of 20 mM and more is established [22–24]. In the rat, lethal plasma lithium levels are reported to be 8 mM. Life threatening effects in humans have been reported with 3 to 4 mM LiCl in serum [33]. Therefore, doses accessible *in vivo* are lower compared to doses used in *in vitro* experiments. Notably, decreased growth of rat mammary carcinoma cells *in vivo* was already obtained after daily injection of 50 mg/kg LiCl [24]. We identified doses between 20.3 mM and 32.1 mM as half maximal inhibitory concentrations for human RMS cell line viability *in vitro*, whereas SKMC control cells exhibited an IC_50_ value of 50.1 mM indicating selectivity. At concentrations between 10 and 25 mM the molecular targets of lithium are GSK-3, but also casein kinase 2 (CK2), MAPKAPK5 and MAPKAPK2 as well as some other non-kinase targets like inositol monophosphatase and histone deacetylase 1 (HDAC1) [32, 34–36]. Of these, GSK-3 is the best-researched target, implicated in the Hh, Wnt and PI3K-PKB pathway which are all involved in RMS biology [11, 37–39]. But also CK2 has been identified as anti-apoptotic factor in the RMS cell lines JR1 and RH-30 [40]. In addition, activation of the MAPK pathway has been implicated in RMS growth [11, 41]. Moreover, inhibition of HDACs has been shown to reduce growth of the ERMS cell line RD and to induce myogenic differentiation [42].

Indeed, we could observe a significant, additive improvement of viability and colony formation reduction as well as induction of cell death upon combined application of ATO and LiCl, compared to single treatment. The viability reduction observed in monolayer cultures could be confirmed in 3D cultures and caspase activation indicated apoptosis initiation. This was accompanied by an inhibitory GSK-3β serine 9 phosphorylation and reduction of GLI1 protein expression in RD and RH-30 cells. Interestingly, SRH cells were more resistant to apoptotic cell death upon ATO and LiCl treatment, whereas additive viability reduction could be documented. While an inhibitory GSK-3β serine 9 phosphorylation was detected in SRH cells after LiCl addition, GLI1 protein expression was clearly enhanced by ATO, LiCl and a combination thereof, indicating that both GSK-3β inhibition and downregulation of GLI1 protein are mandatory for high rates of apoptotic cell death in RMS cell lines. Nevertheless, the mechanism increasing GLI1 abundance in SRH cells remains elusive.

There is an intensive crosstalk between the GLI transcription factors of the Hh pathway and GSK-3 regulating the balance between proliferation, apoptosis and differentiation. Phosphorylation mediated by PKA, GSK-3 and CK1 regulates proteasomal degradation of GLI2 and GLI3 by recruiting β-TRCP [37, 38, 43]. The MEK-RSK cascade positively regulates GLI2 stabilization and represses its degradation via inhibition of GSK-3β-dependent phosphorylation and ubiquitination of GLI2 [44]. Moreover, in murine embryonic fibroblasts and human colon carcinoma cells GSK-3β dependent phosphorylation of GLI3 leads to processing into its transcriptional repressor form GLI3R [45, 46]. Only a minor fraction of murine Gli2 is proteolytically processed to form a transcriptional repressor *in vivo* [47]. A direct phosphorylation of GLI1 by GSK-3 has not been confirmed. However, activation of GLI1 transcriptional activity involves phosphorylation of SUFU by GSK-3 releasing GLI1 from the SUFU complex [48]. Moreover, stabilized β-catenin interacts with GLI1 in murine medulloblastoma spheres, which was increased under lithium treatment mediating GLI1 degradation associated with a G2/M cell cycle arrest and induction of a senescent-like state [17]. In human pancreatic cancer cells lithium treatment reduced the GLI1 mRNA and protein expression [23]. In addition, in A431 epidermoid carcinoma cells and HaCaT immortalized keratinocytes both ATO and LiCl led to GSK-3β inactivation upon serine 9 phosphorylation [25]. LiCl induced apoptotic cell death in human sarcoma and carcinoma cell lines was accompanied by TNF-α and FasL expression inducing PARP and caspase 3, 8 and 10 cleavage [24]. Interestingly, the GSK-3 inhibitors LiCl and SB216763 attenuated caspase 3 and PARP cleavage induced by arsenite (NaAsO2) in SH-SY5Y neuroblastoma cells, indicating that active GSK-3 is required for arsenite induced apoptotic cell death in these cells [49]. Furthermore, it was demonstrated that 6-bromoindirubin-3’-oxime (BIO), a GSK-3 inhibitor, induces Wnt/β-catenin dependent terminal myogenic differentiation of RD, 381T, Rh6 and Rh18 ERMS cell lines, whereas the ARMS cell lines Rh3, Rh5, Rh7 and RH-30 remained in an undifferentiated state upon BIO treatment [39]. In the ARMS cell line RH-30 transcription factors associated with terminal myogenic differentiation like MyoD and myogenin are expressed but inactive. The posttranslational repression of myogenin activity is due to sustained GSK-3β activity leading to inactivation of myogenin trans-activation properties. PAX3-FKHR enhanced the GSK-3β activity and the number of proliferative RH-30 cells was approximately halved by pharmacological inhibition of GSK-3β [50]. Zeng et al. showed that GSK-3 inhibitors, including TWS119 were significantly more effective at inhibiting cell growth and inducing apoptosis in ARMS cell line RH-30 than ERMS cell line RD. GSK-3 inhibitors did not affect the nuclear localization of PAX3-FKHR, but inhibited the transcriptional activity of PAX3-FKHR by phosphorylation [14].

## Conclusion

In summary, we showed additive effects of LiCl and ATO on viability reduction, decrease of colony formation as well as cell death induction in RMS cell lines. This was accompanied by LiCl induced inhibitory GSK-3β serine 9 phosphorylation, whereas GLI1 protein expression was particularly reduced by combined ATO and LiCl treatment in RD and RH-30 cell lines, showing high rates of apoptotic cell death. Therefore, a combination of ATO and LiCl or another drug targeting GSK-3 may be feasible to improve treatment efficiency in RMS.

## Supporting information

S1 Fig Determination of LiCl IC_50_ values by nonlinear regression. MTS assays were performed four days after treatment with LiCl in three RMS cell lines and SKMC in quadruplicate. IC_50_ values were determined by nonlinear regression of MTS results using GraphPad Prism 6. The top was set to 100%, the 95% confidence band was plotted in the graphs.
(TIF)

S2 Fig Determination of ATO IC_50_ values by nonlinear regression. MTS assays were performed four days after treatment with ATO in three RMS cell lines in quadruplicate. IC_50_ values were determined by nonlinear regression of MTS results using GraphPad Prism 6. The top was set to 100%, the 95% confidence band was plotted in the graphs. ATO IC_50_ values have been already published in [9], however the corresponding graphs have not been shown in that publication.
(TIF)

S3 Fig Combination of LiCl and ATO reduces GLI1 abundance in the RMS cell lines RD and RH-30. Western blot analysis with antibodies against GLI1 and PCNA as loading control was performed after 36 h of single or combined treatment with 1 μM ATO (A1), 25 μM LiCl (Li25) and 2 μM itraconazole (I2) in three RMS cell lines in triplicate. The Western blot depicted in the main manuscript contains no itraconazole data. Signals from two additional, independent experiments (2, 3) were quantified to obtain the mean values and standard deviations of full length GLI1 abundance after treatment with ATO and LiCl compared to mock treated control shown in the graph of the main manuscript. Lanes used for quantification in the main manuscript are marked by a black font, lanes not considered in the main manuscript are marked in light grey.
(TIF)

## Abbreviations

APL: acute promyelocytic leukemia
ATO: arsenic trioxide
CK: casein kinase
FasL: Fas ligand
GLI: glioma associated oncogene family
GSK-3: glycogen synthase kinase-3
HDAC: histone deacetylase
Hh: hedgehog
LiCl: lithium chloride
MAPKAPK: MAP kinase activated protein kinase
MEK: mitogen-activated protein kinase kinase
MyoD: myogenic differentiation
PARP: poly (ADP-ribose) polymerase
PAX3-FKHR: paired box 3/ forkhead transcription factor
PCNA: proliferating cell nuclear antigen
PI3K: phosphatidylinositol 3-kinase
PKA: protein kinase A
PKB: protein kinase B
RMS: rhabdomyosarcoma
RSK: ribosomal S6 kinase
SKMC: primary skeletal muscle cells
SMO: smoothened
SUFU: suppressor of fused
TNF-α: tumor necrosis factor α
