# Supplementary figures and images for: Combined application of arsenic trioxide and lithium chloride augments viability reduction and apoptosis induction in human rhabdomyosarcoma cell lines
Source: PLoS One. 2017 Jun 2;12(6):e0178857. doi: 10.1371/journal.pone.0178857 (PMC5456379; doi:10.1371/journal.pone.0178857)

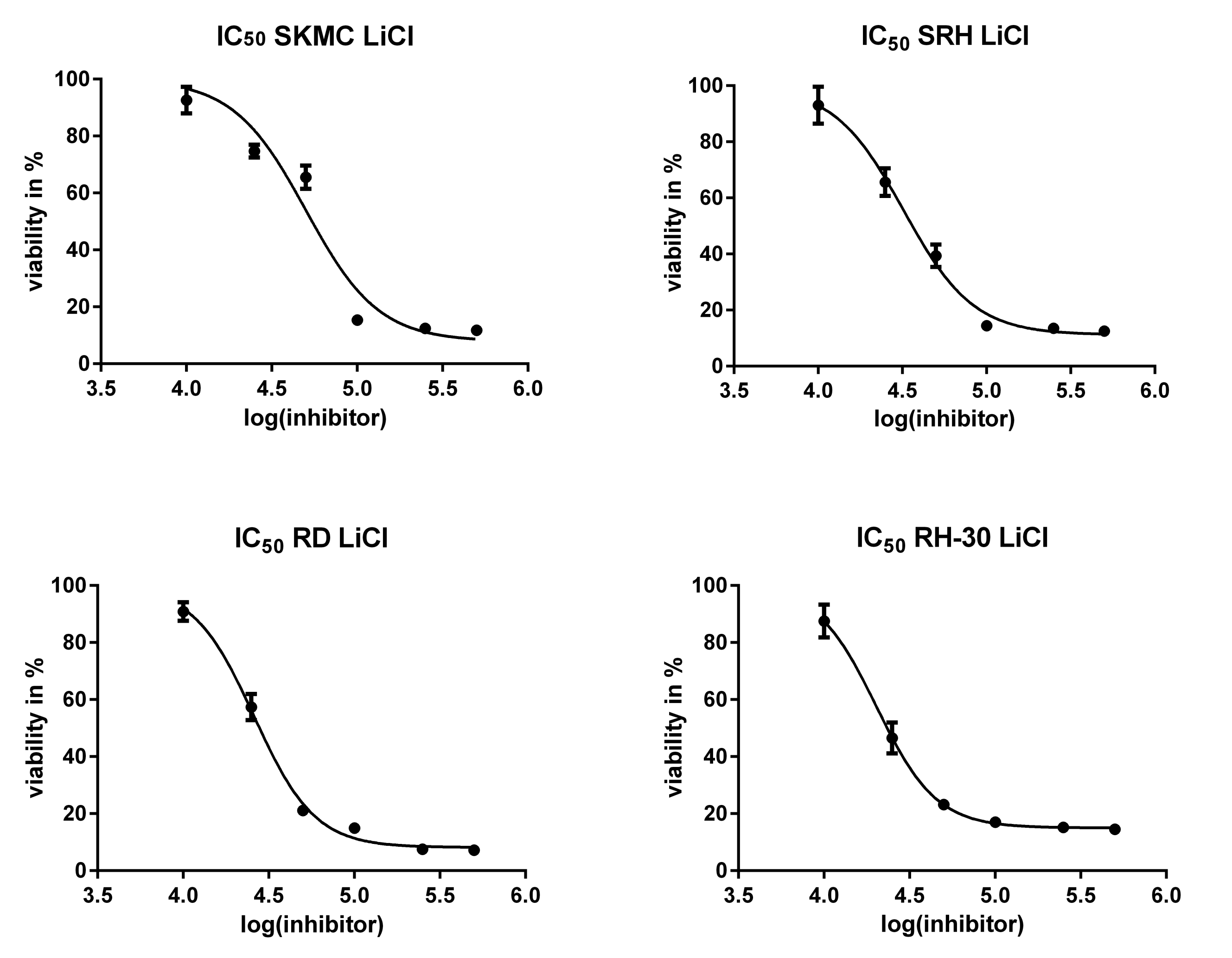

Supplement: S1 Fig — MTS assays were performed four days after treatment with LiCl in three RMS cell lines and SKMC in quadruplicate. IC50 values were determined by nonlinear regression of MTS results using GraphPad Prism 6. The top was set to 100%, the 95% confidence band was plotted in the graphs. (TIF) [file pone.0178857.s001.tif]

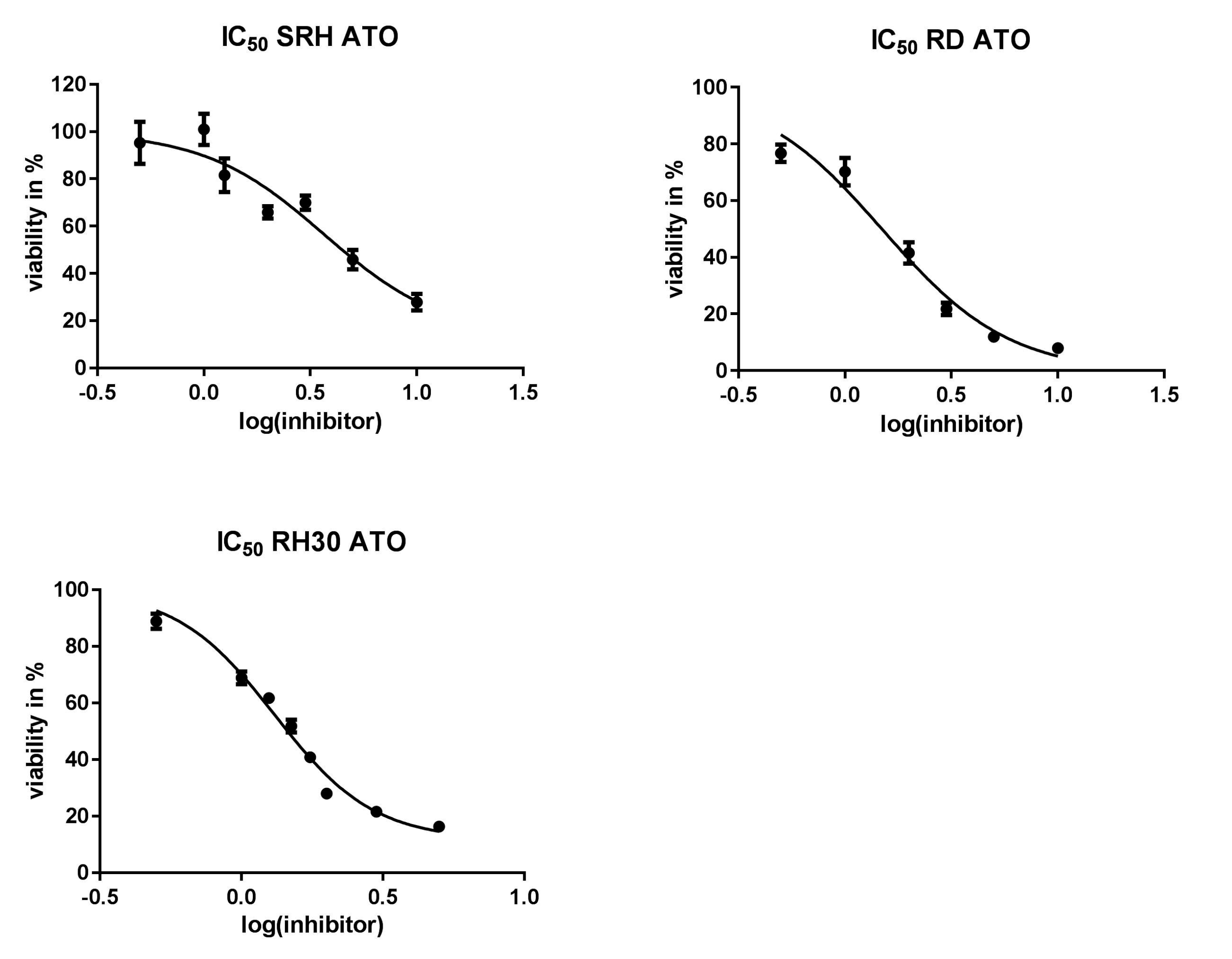

Supplement: S2 Fig — MTS assays were performed four days after treatment with ATO in three RMS cell lines in quadruplicate. IC50 values were determined by nonlinear regression of MTS results using GraphPad Prism 6. The top was set to 100%, the 95% confidence band was plotted in the graphs. ATO IC50 values have been already published in [9], however the corresponding graphs have not been shown in that publication. (TIF) [file pone.0178857.s002.tif]

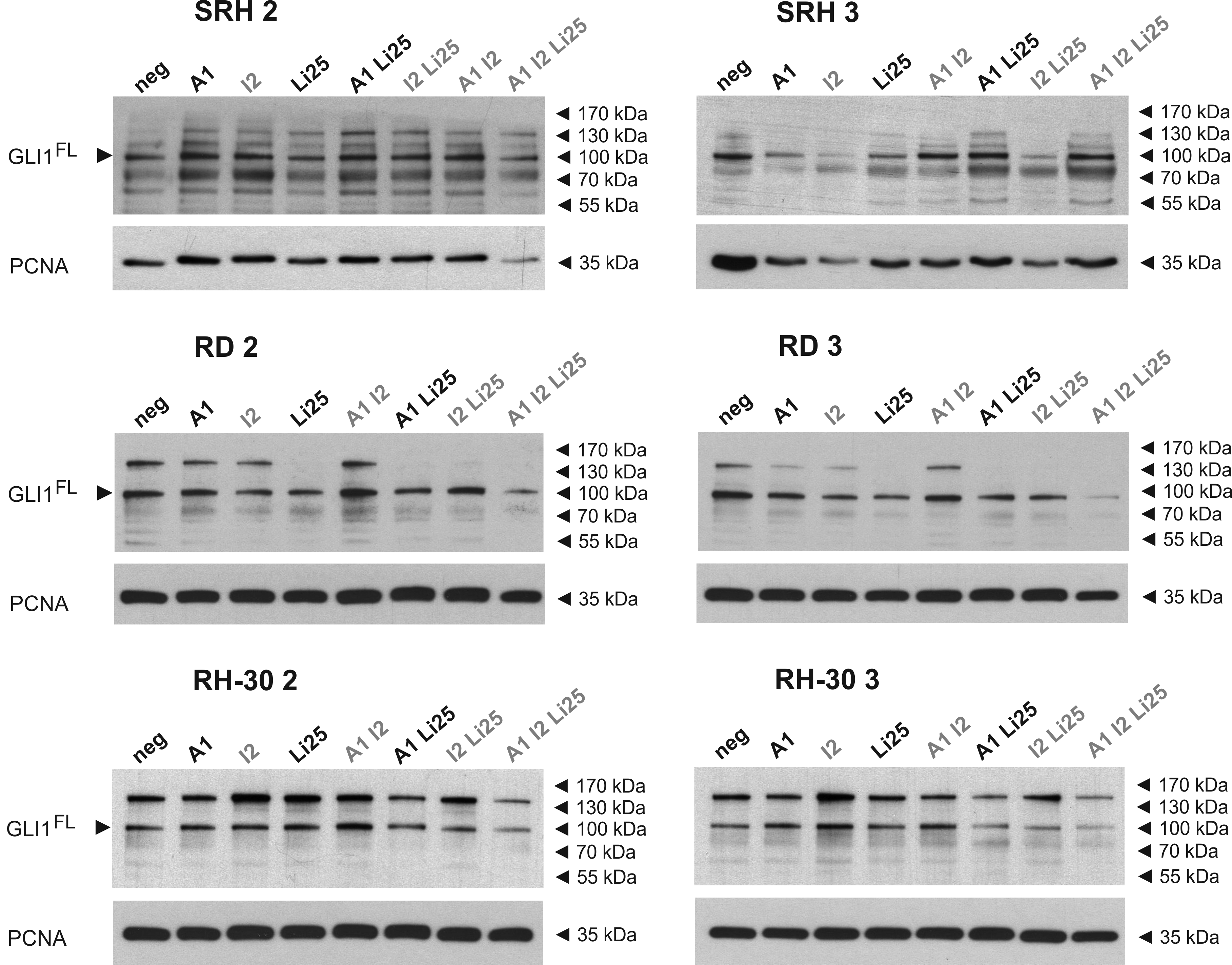

Supplement: S3 Fig — Western blot analysis with antibodies against GLI1 and PCNA as loading control was performed after 36 h of single or combined treatment with 1 μM ATO (A1), 25 μM LiCl (Li25) and 2 μM itraconazole (I2) in three RMS cell lines in triplicate. The Western blot depicted in the main manuscript contains no itraconazole data. Signals from two additional, independent experiments (2, 3) were quantified to obtain the mean values and standard deviations of full length GLI1 abundance after treatment with ATO and LiCl compared to mock treated control shown in the graph of the main manuscript. Lanes used for quantification in the main manuscript are marked by a black font, lanes not considered in the main manuscript are marked in light grey. (TIF) [file pone.0178857.s003.tif]
